# Impact of US Regulatory Approval of Transcatheter Devices to Address Tricuspid Regurgitation: A Single-Center Analysis

**DOI:** 10.1016/j.shj.2026.100819

**Published:** 2026-02-12

**Authors:** Santiago Garcia, Richard Bae, Nadia El-Hangouche, Raviteja R. Guddeti, Puvi Seshiah, Caitlin Stultz, Geoffrey Answinni, Saad Hasan, J. Michael Smith, Abigail de la Rosa, Lynelle M. Logan, Dean J. Kereiakes

**Affiliations:** The Christ Hospital Network and Lindner Center for Research and Education, Cincinnati, Ohio, USA

**Keywords:** Transcatheter tricuspid valve repair, Transcatheter tricuspid valve replacement, Tricuspid regurgitation

## Abstract

• The first year after regulatory approval of transcatheter tricuspid repair and replacement devices, we observed a significant increase in tricuspid regurgitation referrals, advanced cardiac imaging testing, and procedural volumes.

Tricuspid regurgitation (TR) is highly prevalent in the United States.^1^ In 2024, the U.S. Food and Drug Administration approved the first transcatheter devices to repair or replace the tricuspid valve in patients with severe symptomatic TR despite optimal medical therapy.^2^, ^3^, ^4^, ^5^

The goal of this investigation is to examine changes to structural heart disease (SHD) clinic, advanced cardiac imaging, and procedural volumes after U.S. regulatory approval of transcatheter tricuspid repair and replacement devices.

We conducted a single-center analysis of a large and multidisciplinary SHD program in the Midwest region of the United States. The SHD program is part of the Heart and Vascular Institute at The Christ Hospital Network (Cincinnati, OH), a group with >120 providers, including cardiologists, cardiac and vascular surgeons, and advanced practice providers serving the tristate region of Southern Ohio, Northern Kentucky, and Southeast Indiana.

We analyzed SHD clinic, advanced cardiac imaging, and procedural volumes per valve and by consultation status (new vs. existent) for fiscal year (FY) 2023-2025. Each FY starts on July 1 of the previous calendar year and extends to June 30 of the next calendar year.

The EVOQUE transcatheter tricuspid valve replacement system (Edwards Lifesciences, Irvine, California) was approved on February 1, 2024.^3^ The TriClip transcatheter tricuspid valve repair system (Abbott Medical, St. Paul, Minnesota) was approved on April 1, 2024.^2^ Therefore, both devices were approved in FY 2024. For the study, FY 2023 was considered preapproval and FY 2025 was considered postapproval. Although research cases were not included in the present analysis, the SHD program at The Christ Hospital participated in both pivotal trials and continued access registries, and therefore, had access to both technologies immediately after commercial approval.

Clinical and procedural volumes were obtained from financial claims data. As financial claims data may not capture some procedures (e.g., inpatient, prolonged hospital stays with a non-cardiology physician as the primary attending), whenever possible, procedural volumes were verified by a second data source (quality reporting system to Society of Thoracic Surgeon [STS]/American College of Cardiology [ACC] Transcatheter Valve Therapy [TVT] Registry). Discrepancies were resolved by consensus. All structural imaging tests (computed tomography [CT] and transesophageal echocardiograms [TEE]) are performed and analyzed by a dedicated group of structural imagers. Any CT or TEE with a diagnosis of valvular heart disease interpreted by one of the structural imagers was considered a structural diagnostic imaging test. New clinic encounters were stratified by valve type (aortic, mitral, and tricuspid). Encounters that did not fit a specific valve type (heart failure devices, left atrial appendage occlusion, patent foramen ovale, atrial septal defect, transcatheter pulmonary intervention, etc.) were categorized as others.

During the study period, we observed an 11% growth in SHD clinic volumes from 2317 encounters in FY 2023 to 2585 encounters in FY 2025 (Figure 1a). This growth was primarily driven by new SHD outpatient encounters that increased from 355 in FY 2023 to 448 in FY 2025 (26% growth). Established SHD clinic encounters increased from 1962 in FY 2023 to 2137 in FY 2025 (8% growth). When new clinic encounters were analyzed by valve type (Figure 1b), we observed a 16% increase in aortic referrals, which included both aortic stenosis and regurgitation, whereas outpatient new mitral referrals remained stable (FY 2023: 93, FY 2024: 101, FY 2025: 93). New tricuspid outpatient referrals grew 221% from 28 in FY 2023 to 62 in FY 2025. The number of SHD TEE increased 78% during the study period from 293 in FY 2023 to 523 in FY 2025. Similarly, the number of SHD CT angiograms increased 46% from 530 in FY 2023 to 778 in FY 2025.

**Figure 1:**
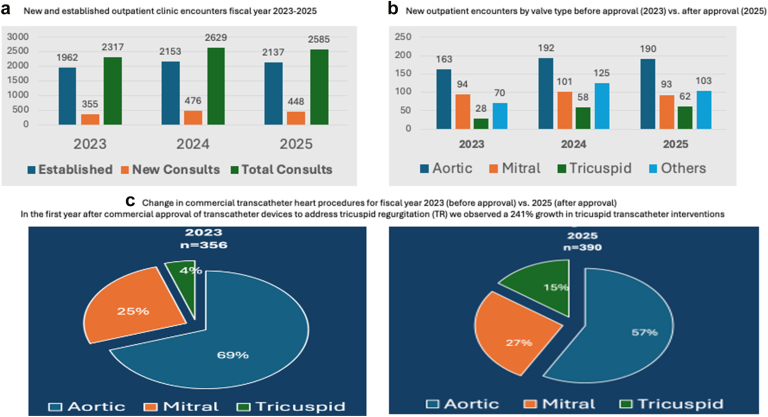
(a) New and established outpatient clinic encounters for the structural heart program during fiscal year 2023-2025. (b) New outpatient encounters by valve type before approval (2023) vs. after approval (2025). (c) Change in commercial transcatheter heart procedures before approval (2023) vs. after approval (2025).

Overall volume of commercial transcatheter valve procedures, including repair and replacement, increased 10% during the study period from 356 in FY 2023 to 390 in FY 2025. However, differential patterns of growth were seen according to valve type (Figure 1c). Tricuspid procedures increased 241% from 17 in FY 2023 to 58 in FY 2025. Mitral procedures increased 20% from 92 in FY 2023 to 106 in FY 2025. Commercial transcatheter aortic valve replacement (TAVR) procedures decreased by 8% from 247 in FY 2023 to 229 in FY 2025. Aggregated mitral-tricuspid procedures increased from 109 in FY 2023 to 164 in FY 2025. When TAVR cases with dedicated devices for aortic regurgitation were included (research cases), overall TAVR volumes were stable (252 TAVR cases in FY 2023 and 244 in FY 2025, - 3%).

We conducted a single-center analysis of outpatient clinic encounters, dedicated SHD imaging tests, and procedural volumes after US commercial approval of transcatheter devices for patients with severe, symptomatic TR despite OMT. Several key findings are noted. First, there has been significant growth in the number of TR referrals, associated imaging tests, and procedural volumes. Second, the proportion of mitral-tricuspid procedures as a percentage of overall transcatheter valve volume has increased from 29% in FY 2022 to 43% in FY 2025. Given the resource intensive nature of mitral-tricuspid transcatheter procedures—which in most cases require preprocedural TEE, structural CT angiography, right heart catheterization, and are mostly performed under general anesthesia—we believe these trends have profound implications for SHD programs. For SHD programs performing (or considering performing) transcatheter tricuspid valve interventions, adequate resource planning, allocation, and workflow efficiencies are essential to accommodate and sustain growth.

Our study should be interpreted considering two limitations. First, inpatient consultations and research cases were not included in the analysis. Second, the number of SHD programs performing transcatheter tricuspid valve replacement procedures still represents a small fraction of TAVR programs. Therefore, our results may not be generalizable to other SHD programs yet.

In the first year after commercial introduction of transcatheter tricuspid repair and replacement devices, we observed a significant increase in TR referrals, advanced cardiac imaging tests, and procedural volumes. These results have implications for other SHD programs in terms of resource planning, allocation, and workflow efficiencies.

## Ethics Statement

The research reported adheres to ethical guidelines.

## Funding

The authors have no funding to report.

## Disclosure Statement

Santiago Garcia reports consultant fees from Edwards Lifesciences, Medtronic, Abbott Vascular, Boston Scientific, Anteris, Capstan Medical, B. Braun; proctoring for Edwards Lifesciences and Abbott Vascular; advisory board participation for Medtronic and Boston Scientific; stock options for Capstan Medical. Richard Bae is a consultant and proctor for Abbott Vascular. The other authors have nothing to disclose.
